# The Cell Activation Phenomena in the Cold Atmospheric Plasma Cancer Treatment

**DOI:** 10.1038/s41598-018-33914-w

**Published:** 2018-10-18

**Authors:** Dayun Yan, Wenjun Xu, Xiaoliang Yao, Li Lin, Jonathan H. Sherman, Michael Keidar

**Affiliations:** 1 Department of Mechanical and Aerospace Engineering, The George Washington University, Science & Engineering Hall, 800 22nd Street, NW, Washington, DC 20052 USA; 2 State Key Laboratory of Electrical Insulation and Power Equipment, Xi’an Jiaotong University, Xi’an, 710049 China; 3 Neurological Surgery, The George Washington University, Foggy Bottom South Pavilion, 22nd Street, NW, 7th Floor, Washington, DC 20037 USA

## Abstract

Cold Atmospheric Plasma (CAP) is an ionized gas with a near room temperature. CAP is a controllable source for reactive species, neutral particles, electromagnetic field and UV radiation. CAP showed the promising application in cancer treatment through the demonstration *in vitro* and *in vivo*. In this study, we first demonstrate the existence of an activation state on the CAP-treated cancer cells, which drastically decreases the threshold of cell vulnerability to the cytotoxicity of the CAP-originated reactive species such as H_2_O_2_ and NO_2_^−^. The cytotoxicity of CAP treatment is still dependent on the CAP-originated reactive species. The activation state of cancer cells will not cause noticeable cytotoxicity. This activation is an instantaneous process, started even just 2 s after the CAP treatment begins. The noticeable activation on the cancer cells starts 10–20 s during the CAP treatment. In contrast, the de-sensitization of activation takes 5 hours after the CAP treatment. The CAP-based cell activation explains the mechanism by which direct CAP treatment causes a much stronger cytotoxicity over the cancer cells compared with an indirect CAP treatment do, which is a key to understand what the effect of CAP on cancer cells.

## Introduction

CAP is an ionized near-room temperature gas, composed of reactive species, neutral particles and molecules, electrons and other physical factors such as electromagnetic field, weak ultraviolet radiation and weak heating effect^1^. CAP has been widely used in many branches of modern medicine, including wound healing and sterilization^2^. Over the past decade, CAP showed the promising application in the cancer treatment^3,4^. CAP can effectively and selectively kill dozens of cancer cell lines *in vitro* through a direct CAP treatment on the cells or through an indirect CAP treatment on the medium or other biologically adaptable solutions which will be further used to affect the growth of cancer cells. In some cases, it is also observed that CAP treatment does not show selective on tumor cells^5^. The growth of nonmalignant cells can also be noticeably affected by the CAP treatment^6^. CAP treatment can also significantly inhibit the growth of xenografted tumors in mice through a direct CAP treatment on the skin above the tumorous tissue^7^. The chemical factors in CAP such as reactive species have been regarded as the main anti-cancer factors during CAP treatment^8,9^. This conclusion is strongly supported by the observation that the CAP-treated medium, a solution containing most long-lived reactive species originated from CAP, can cause similar strong and even selective anti-cancer effect *in vitro* and *in vivo*^10,11^. The long-lived reactive species at least include H_2_O_2_, NO_2_^−^, and NO_3_^−^. The key role of these long-lived reactive species in the cytotoxicity of CAP treatment has been extensively investigated over the past decade^10–12^. Using specific scavengers such as cysteine and catalase in the medium can completely eliminate the cytotoxicity of CAP treatment in many cases, which may be due to the consumption of the CAP-generated ROS or RNS by these scavengers^13,14^. Renewing the medium immediately after CAP treatment will also completely inhibit the killing effect of CAP treatment^15^. Clearly, the cytotoxicity of CAP must be depended on these reactive species. However, these studies give us an impression that a CAP treatment, either the direct treatment or the indirect treatment using the CAP-treated medium, is just a reactive species-based treatment particularly the treatment just based on the long-lived reactive species.

However, the direct CAP treatment should be different from the indirect CAP treatment at least in terms of the short-lived reactive species and potential other effects such as physics effects of CAP treatment on cells. The fact is that the comparison between the direct and indirect CAP treatment under the same experimental condition is rare in plasma medicine. In a few comparison that performed under the same experimental conditions, the direct CAP treatment showed a much stronger killing effect on cancer cells compared with the treatment using the CAP-treated medium^16,17^. The chemical components particularly H_2_O_2_ and NO_2_^−^ in the medium are the same regardless of the chosen treatment strategy, direct or indirect^16^. Therefore, there must be some unknown factors significantly affect the cytotoxicity of the reactive species on cells.

**Figure 1:**
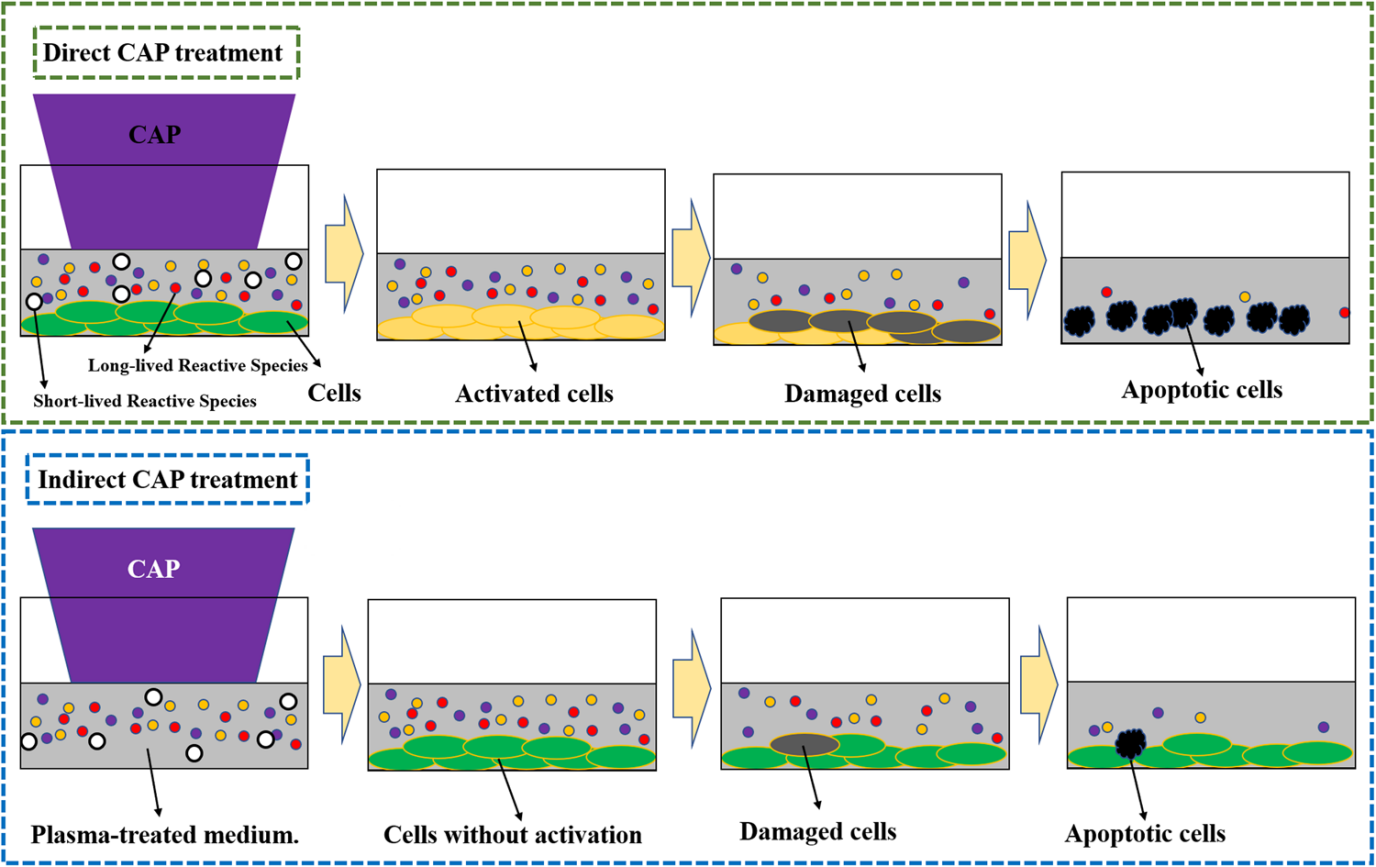
A schematic illustration of the CAP-based cell activation during the direct CAP treatment. In two cases, the concentration of reactive species in the CAP-treated medium is the same. The CAP-treated (activated) cells are sensitive to the cytotoxicity of the CAP-originated reactive species. In contrast, the indirect CAP treatment based on the CAP-treated medium on the cancer cells without the activation will only cause a noticeable weaker cytotoxicity. This model explains the stronger anti-cancer effect of a direct CAP treatment on cancer cells compared with an indirect CAP treatment. A CAP treatment not only provides the dozens of reactive species in the extracellular environment, but also decreases the threshold of the cancer cells to the cytotoxicity of the reactive species through the activation. This is a fundamental feature of the CAP treatment. The activation state of the CAP-treated cancer cells may be due to the short-lived reactive species or other unknown factors.

We propose that there exists an activation state of cells after the direct CAP treatment (Fig. 1). Such an activated state is the unique response of cells to CAP treatment. The activation state of cells will not cause an observable inhibition on the cell growth. It simply activates the CAP-treated cells into a unique state, in which cancer cells are very sensitive to the CAP-originated reactive species. Here, we first demonstrate the experimental evidence about the existence of such activation state of cells after a CAP jet treatment. Even a low concentration of H_2_O_2_, or NO_2_^−^ can cause a noticeable killing effect on the CAP-activated cancer cells, though these reactive species will not cause significant killing effect on the same cancer cells without such activation. This discovery drastically changes our previous understanding of the anti-cancer mechanism of CAP treatment. It should be pointed out that plasma activation reported here is somewhat analogous to sensitizing cells utilized widely in chemotherapy^18^.

## Materials and Methods

### CAP device

The CAP jet device was designed and assembled in Dr. Keidar’s lab. It has been used in many studies *in vitro* and *in vivo*. The detailed description of this device has been illustrated in previous investigation^10,16,19^. The experimental setup is just simply introduced at here. Helium was used as the carrying gas to trigger the discharge process and the formation of CAP jet. The CAP jet was formed through the discharge (Pk-Pk: 5.8 kV) between a ring grounded cathode and a central anode and was flowed out (4.7 L/min) by the carrying gas in a glass tube with a diameter of 4.5 mm. The discharge process was driven by an AC high voltage (3.16 kV) with a frequency of 12.6 kHz.

### Cells culture

Human pancreas adenocarcinoma (PA-TU-8988T) cells were provided by Dr. Murad’s lab at the George Washington University. The Dulbecco’s modified Eagle’s medium (DMEM, 11965-118) was purchased from Life Technologies. DMEM was mixed with 1% (v/v) antibiotic (penicillin and streptomycin) solution (Life Technologies). The media used in cell culture were composed of DMEM supplemented with 10% (v/v) fetal bovine serum (ThermoFisher Scientific). The cells were cultured in a 96-well plate (61406-081, Corning). 100 μL of the cells-harvesting solution (3 × 10^4^ cells/mL) were seeded in each well. Cancer cells were grown for 1 day under the standard culture condition (a humidified, 37 °C, 5% CO_2_ environment). The media that have been used to culture cells overnight were removed before any experiments.

### Making CAP-stimulated medium (PSM), H_2_O_2_/NO_2_^−^/NO_3_^−^-containing DMEM (H_2_O_2_/NO_2_^−^/NO_3_^−^-DMEM) and treating cancer cells

To make PSM, DMEM in multi-well plate was treated by CAP. Then, the CAP-treated DMEM was immediately (<20 s) removed to affect the growth of cancer cells in 96-well plate. To make H_2_O_2_-DMEM, 9.8 M H_2_O_2_ standard solution (Sigma-Aldrich, 216763) were added in DMEM with a designed concentration. Similarly, to make NO_2_^−^-DMEM and NO_3_^−^-DMEM, NO_2_^−^ standard solution (Sigma-Aldrich, 72586) and NO_3_^−^ standard solution (Sigma-Aldrich, 74246) was added in DMEM with a designed concentration, respectively. Then, these DMEM was transferred to affect cancer cells in a 96-well plate. For the control group, cancer cells were just cultured in the untreated DMEM. Cancer cells were cultured 3 days after the treatment. The sample number in the direct CAP treatment and the indirect CAP treatment was 3 and 6, respectively. The sample number in the H_2_O_2_, NO_2_^−^, or NO_3_^−^ treatment was 3. Each experiment was independently repeated for at least three times.

### Cell viability assay

The cell viability was measured by using MTT (3-(4,5-Dimethyl-2-thiazol)-2, 5-Diphenyl-2H-tetrazolium Bromide) assay according to the standard protocols provided by the manufacturer (Sigma-Aldrich, M2128). The 96-well plate was read by an H1 microplate reader (Hybrid Technology) at the absorbance of 570 nm after a 30 s of shaking in the reader. To facilitate the data analysis, the measured absorbance at 570 nm was processed to be a relative cell viability by the division of absorbance between the experimental group and the control group. In the figures, the relative cell viability was expressed as ‘cell viability (fold)”.

## Experiments and Results

**Figure 2:**
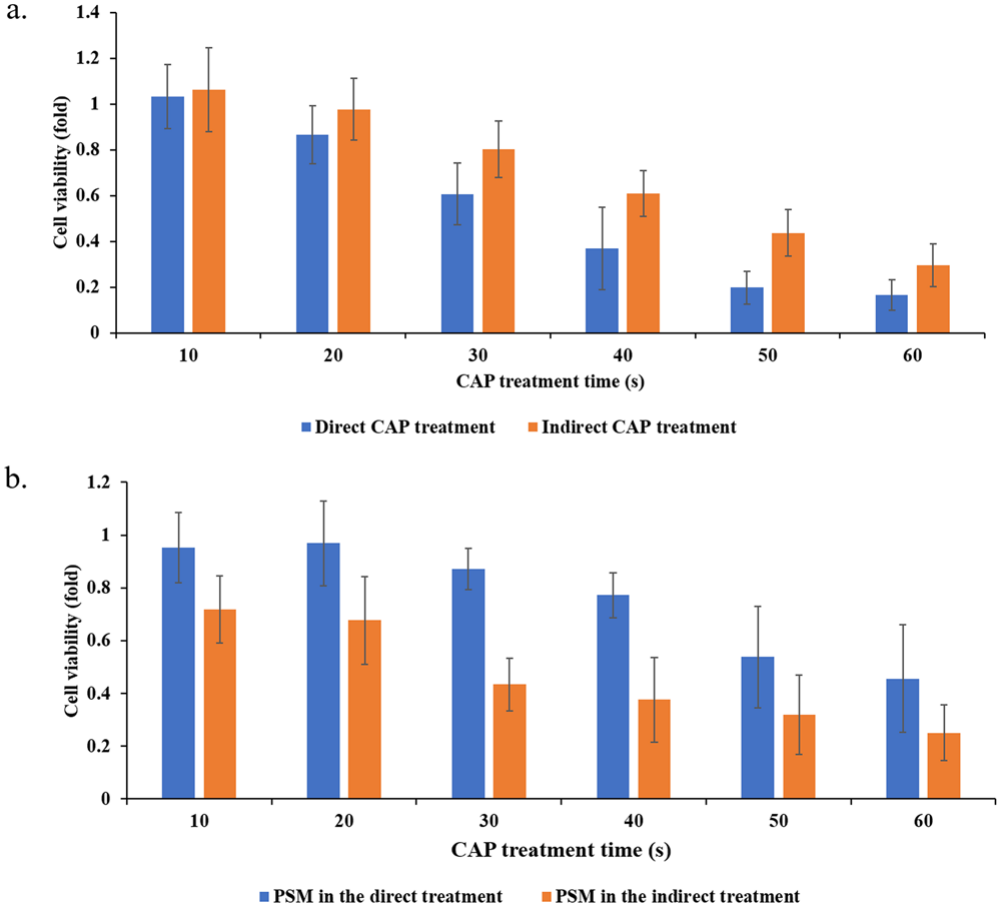
The reactive species cannot explain the cytotoxicity of a direct CAP treatment on pancreatic adenocarcinoma cell line PA-TU-8988T. (**a**) A direct CAP treatment caused a stronger killing effect on PA-TU-8988T cells than an indirect CAP treatment under the same experimental conditions. The direct CAP treatment was performed by treating cancer cells on a 96-well plate. These cells were immersed in 50 μL DMEM. The indirect CAP treatment was performed by treating 50 μL DMEM in a 96-well plate. The 50 μL of treated DMEM was immediate transferred to affect the growth of cancer cells in an another 96-well plate. (**b**) Unknown cell-based protective factors existed in the medium after the direct CAP treatment. The direct CAP treatment was performed by treating the cancer cells immersed in 55 μL DMEM on a 96-well plate. After the treatment, 50 μL of these DMEM was immediately (<30 s after the treatment) transferred to affect the cancer cells grown in a new 96-well plate. These DMEM contains the CAP-originated reactive species and all known cell-based production during the CAP treatment. In contrast, the indirect CAP treatment was performed by treating 55 μL DMEM in a 96-well plate without touching any cells. 50 μL of these DMEM was immediately (<30 s after the treatment) transferred to affect the new cancer cells grown in a new 96-well plate. These DMEM just contained the CAP-originated reactive species. All cells were cultured 3 days before the cell viability assay. All experiments were performed in triplicate and were independently repeated for at least three times. The results are shown as the mean ± standard deviation (sd).

The indirect CAP treatment has shown a strong anti-cancer effect on several cancer cell lines *in vitro* and subcutaneously xenografted tumors *in vivo*. However, a comparison between the direct CAP treatment and the indirect CAP treatment under the same experimental conditions shows that a direct CAP treatment will result in a much stronger cytotoxicity (Fig. 2a). The detailed experiment protocols were illustrated in the legend. This trend has also been observed in our previous report and many other studies^16,17^. Many reactive species are formed in the CAP-treated medium. Other unknown factors may also affect the cytotoxicity of CAP treatment on cells. To get a complete picture about the instantaneous change in the CAP-stimulated medium (PSM) with or without touching cells during the CAP treatment, we further investigated the cytotoxicity of PSM made in the direct CAP treatment and in the indirect CAP treatment. The detailed protocols were illustrated in the legend. The medium after the indirect CAP treatment will only contain the CAP-originated reactive species. In contrast, the medium after the direct CAP treatment will contain not only these reactive species but also all cell-related products upon the CAP treatment. One example is the cell-based H_2_O_2_ generation during the CAP treatment^16^. It is found that the medium after the direct CAP treatment showed a weaker cytotoxicity than the medium after the indirect CAP treatment (Fig. 2b). Our previous studies found that the instantaneous H_2_O_2_ concentration in the CAP-treated medium would be noticeably increased due to the appearance of cancer cells during the CAP treatment^16^. Though the decomposition of ROS by cancer cells during the CAP treatment may noticeably exist, the experimental results demonstrated that at least H_2_O_2_ did not show degradation during the CAP treatment process. Thus, some unknown protective factors might be generated during the direct CAP treatment.

Clearly, the reactive species or other factors in medium could not explain the trend shown in Fig. 2a. It is reasonable to assume that there must be a third factor to cause the stronger cytotoxicity of a direct CAP treatment on pancreatic cancer cells compared with the case using the indirect CAP treatment.

We proposed that there was an activation state of cancer cells after the direct CAP treatment, which is the primary reason for the strong cytotoxicity of CAP treatment even though the concentration of reactive species is not so high. Such activation state sensitized the cancer cells into a specific state, in which the cancer cells were very vulnerable to the cytotoxicity of reactive species including ROS and RNS. To demonstrate the existence of such activation state, we first investigated the cytotoxicity of three main reactive species (H_2_O_2_, NO_2_^−^, and NO_3_^−^) and the CAP-treated medium on PA-TU-8988T cells. We displayed that only the H_2_O_2_-containing DMEM could cause significant and even complete growth inhibition on the cell viability of PA-TU-8988T cells (Supporting Materials, Fig. S1a). The growth inhibition has not been observed even though the concentration of NO_2_^−^ or NO_3_^−^ increases to 90 μM (Supporting Materials, Fig. S1b,c).

To clearly observe the activation state of cancer cells, the cytotoxicity of these reactive species on the cancer cells without the activation should not be so strong. The activation state will be observed when a cytotoxicity of these reactive species on the CAP-activated cancer cells is stronger than that of the same reactive species on the cancer cells without a CAP treatment. Thus, we intentionally prepared these reactive species-containing DMEM without a relative low concentration to reveal the activation state.

**Figure 3:**
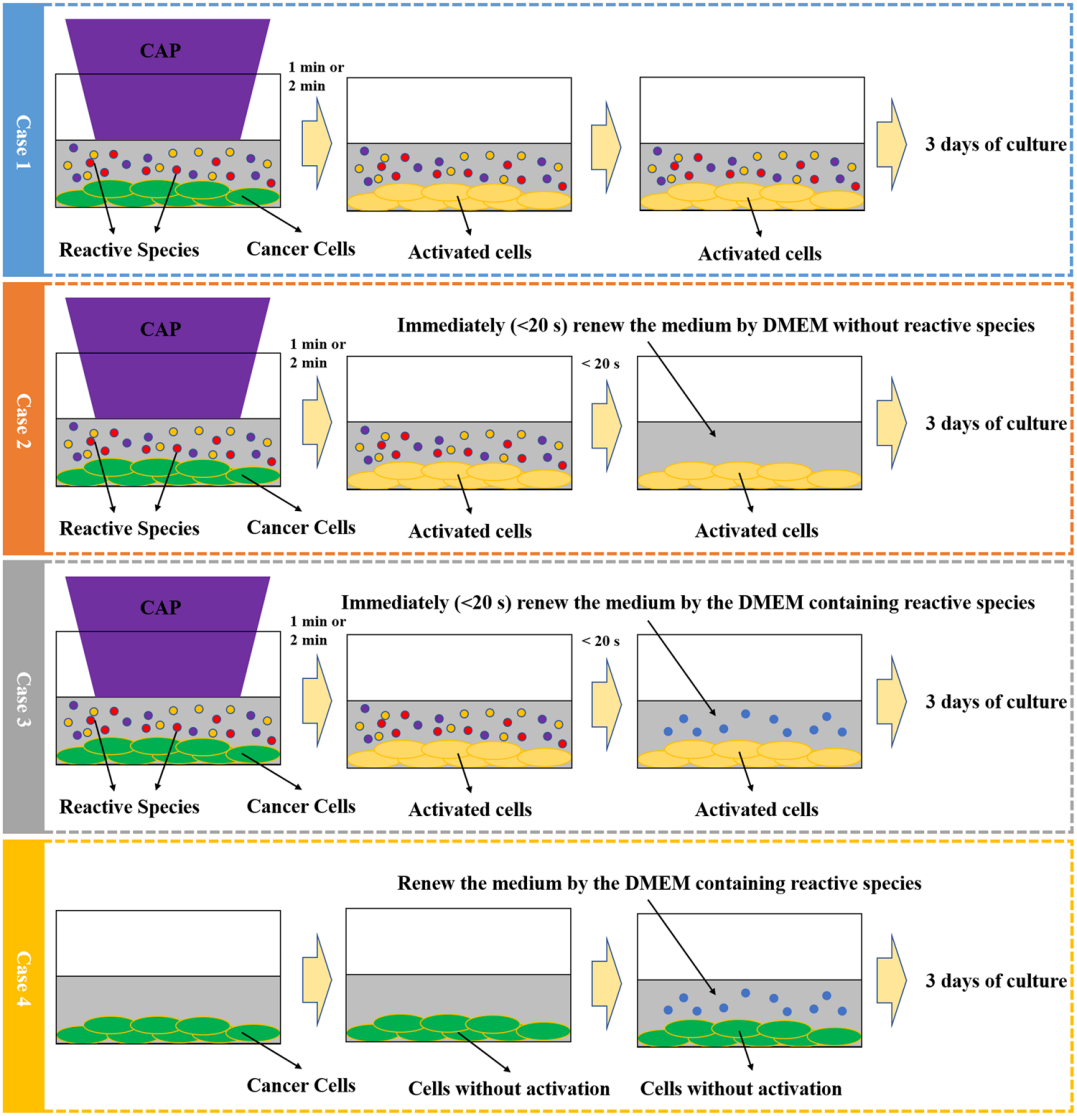
The research strategy to demonstrate the existence of the activation state. The activation state of the cancer cells was achieved by a direct CAP jet treatment. A quick (<20 s after the CAP treatment) removal of the medium after the CAP treatment was used to eliminate the effect of long-lived reactive species in the medium. The quick removal of the medium sustained the cell’s growth and retained the activation state of cancer cells. The comparison between the cytotoxicity of the reactive species-based treatment such as a H_2_O_2_ treatment on the cancer cells activated by CAP jet and that on the cancer cells without the CAP activation revealed the existence of the activation state. The protocols in each case was the same throughout the whole manuscript. Case 1, Case 2, Case 3, and Case 4 responded to ‘One step CAP treatment’, ‘Activated cells cultured in DMEM without reactive species’, ‘H_2_O_2_ treatment on the cells with activation’, and ‘H_2_O_2_ treatment on the cells without activation’, respectively.

To that end, we developed a novel research strategy, which has not been used in previous studies. The research strategy was based on four basic experimental cases, which served as the framework of the protocol (Fig. 3). Case 1: The direct CAP treatment was performed on a well of a 96-well plate. The cancer cells were immersed in 50 μL of DMEM. In this experiment, the cancer cells were activated by the CAP jet. The reactive species from CAP were accumulated in DMEM to further affect cancer cells in the following 3 days of culturing. Case 2: The direct CAP treatment was performed first similar to the Case 1. After that, 50 μL of DMEM was quickly (<20 s after the CAP treatment) removed and was renewed by the 50 μL of new DMEM. In this experiment, the cancer cells were still activated by the CAP jet. However, the reactive species generated by CAP were removed. Thus, the effect of CAP-originated reactive species could be neglected in this case. Case 3: The cancer cells were treated by the CAP jet first. The medium was also immediately (<20 s after the CAP treatment) removed. After that, a 50 μL of reactive species-containing DMEM such as H_2_O_2_ - containing DMEM, NO_2_^−^-containing DMEM, NO_3_^−^-containing DMEM, or the CAP-treated DMEM, was used to affect the activated cancer cells. Case 4: Without the CAP treatment, these cancer cells were cultured in a 50 μL of reactive species-containing DMEM such as H_2_O_2_ - containing DMEM after the initial 50 μL medium was removed. The control in each case has not been shown here. The final cell viability (fold) was obtained by the division of the absorbance between the experimental group and the control group. All these cells were cultured for 3 days before the final cell viability assay. The cell viability was obtained using the methods mentioned in Materials and Methods.

**Figure 4:**
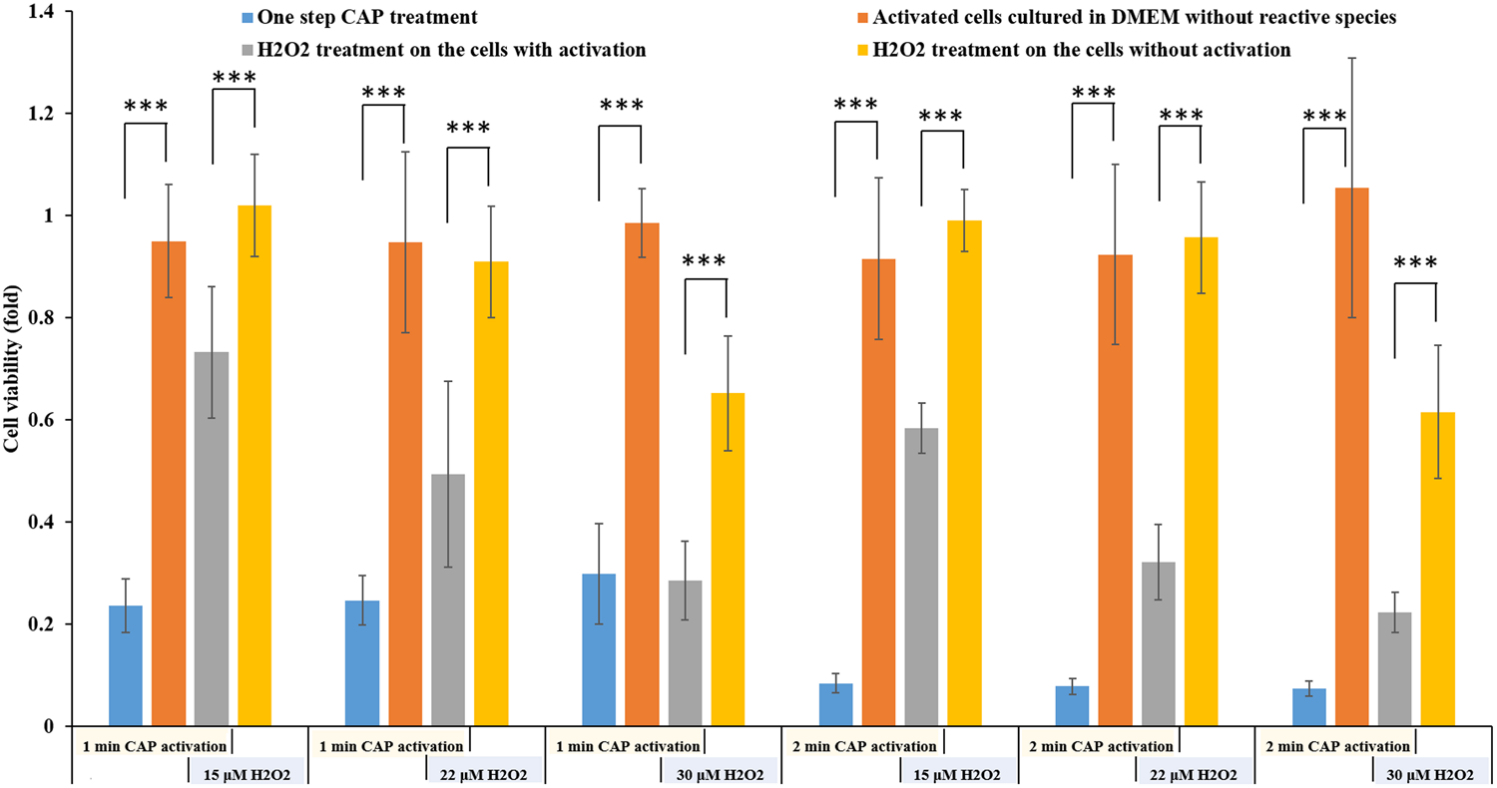
The demonstration of the existence of the activation state after the direct CAP treatment. The activation state of cells will significantly weaken the threshold of cancer cells to the cytotoxicity of H_2_O_2._ The “1 or 2 min CAP activation” means that a direct CAP treatment was performed on cancer cells in 96-well plate for 1 min or 2 min. The “15, 22, or 30 μM H_2_O_2_” means that a specific H_2_O_2_ concentration of H_2_O_2_-DMEM is used to affect the growth of the cancer cells with or without an activation. The data of Case 1, Case 2, Case 3, and Case 4 were shown in blue, orange, gray, and yellow, respectively. The protocols were illustrated in text. All experiments were performed in triplicate and were independently repeated for at least 3 times. The results were shown as the mean ± sd. Student’s t-tests were performed and the significance was indicated as ***p < 0.005, **p < 0.01, *p < 0.05, respectively.

We first demonstrated that the pancreatic cancer cells could be activated into a sensitized state, in which the CAP-treated cancer cells were much more sensitive to the reactive species compared with the cancer cells without such CAP treatment. First, the reactive species were necessary for the cytotoxicity of CAP treatment, though it could not explain the cytotoxicity of the direct CAP treatment. The direct CAP treatment caused a noticeable damage on PA-TU-8988T cells (blue bars in Fig. 4). The H_2_O_2_ generation in 96-well plate was 26.7 μM after a 1 min of CAP treatment (Supporting Materials, Fig. S2a). According to the data in Supporting Materials, Fig. S1a, the H_2_O_2_ with such concentration only caused about a 40% killing effect on PA-TU-8988T cells. In contrast, a 1 min of CAP treatment caused about 80% killing effect on cancer cells (blue bars in Fig. 4). In addition, a quick removal of the medium after the CAP treatment nearly completely counteracted the cytotoxicity of the CAP treatment on PA-TU-8988T cells (orange bars in Fig. 4). As we proposed above, the activation state would not cause noticeable damage on cancer cells studied. Recall that a quick removal of the medium cannot 100% counteract the cytotoxicity of a CAP treatment lasting adequately long. Thus, the CAP jet might cause other cellular damage to cancer cells just during a 1 min or 2 min of treatment. Such damage was not reversable and was different from the effect related to the activation state.

When we used H_2_O_2_ to treat these CAP-treated cancer cells, the strong sensitivity of these cells to the cytotoxicity of H_2_O_2_ was first demonstrated. The H_2_O_2_ – DMEM (15 μM and 22 μM) did not cause strong killing effect on PA-TU-8988T cells without an activation (yellow bars in Fig. 4). In contrast, the same H_2_O_2_ treatment on the CAP-activated PA-TU-8988T cells will cause a strong killing effect (gray bars in Fig. 4). For example, the killing effect of 22 μM H_2_O_2_ on the cancer cells activated by CAP and the cancer cells without activation was about 10% (yellow bar) and 50% (gray bar), respectively. Similar trend was observed on other H_2_O_2_ treatments in Fig. 4. We named such enhanced sensitivity or vulnerability during the CAP treatment as ‘the activation of cells due to CAP treatment’. It is not only a new conception in plasma medicine, but also a novel cellular response to the CAP treatment.

**Figure 5:**
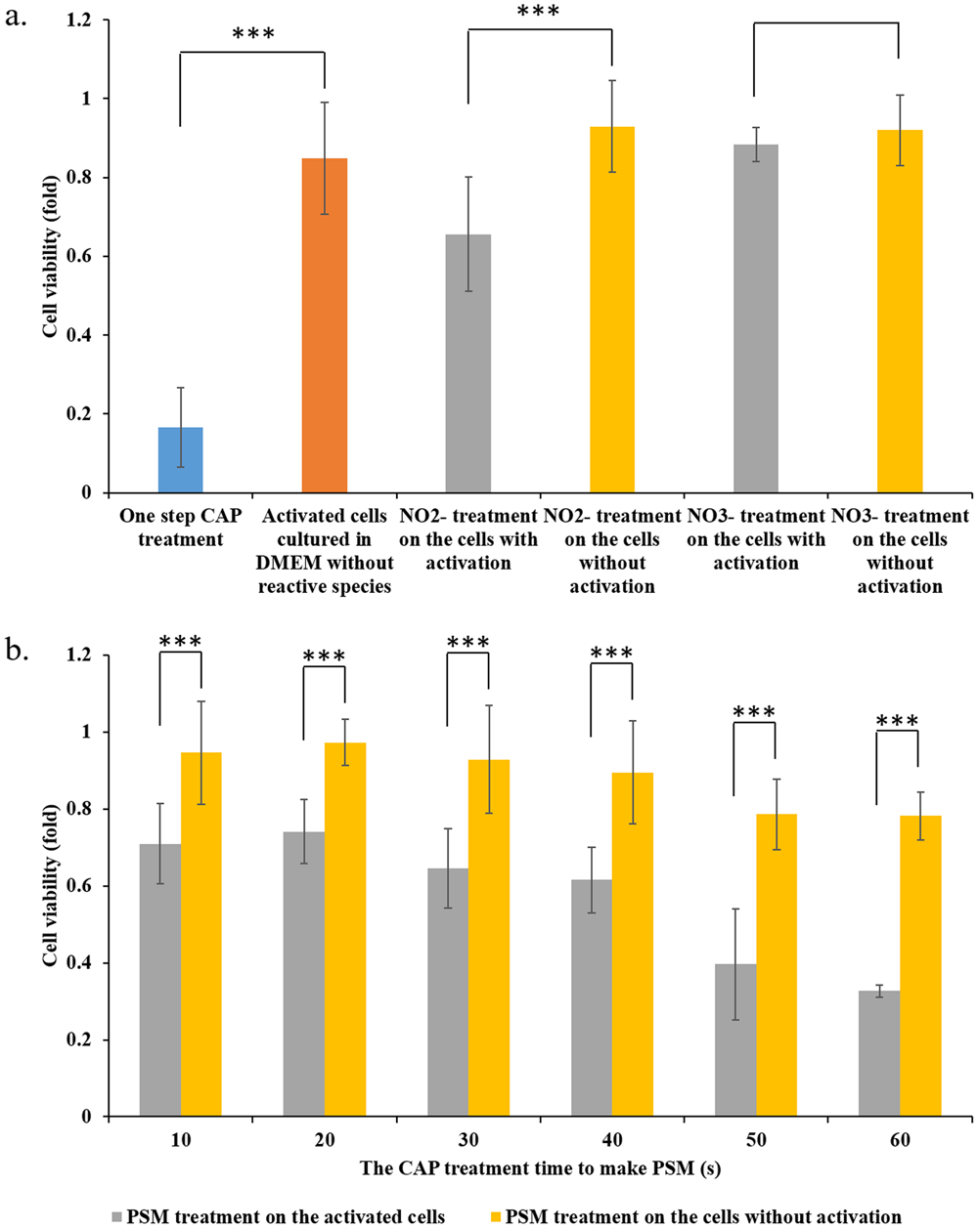
CAP can activate the cancer cells into a sensitized state, in which the cells are very sensitive to the reactive species. (**a**) The cytotoxicity of NO_2_^−^ (50 μM) and NO_3_^−^ (50 μM) on the activated cancer cells. (**b**) The cytotoxicity of PSM on the activated cancer cells. The basic research strategy was the same as that illustrated in Fig. 3. The protocols to prepare the NO_2_^-^DMEM and NO_3_^-^DMEM were illustrated in Materials and Methods. PSM was made by treating DMEM (1 mL/well) in 12-well plate by the CAP jet following the method in Materials and Methods. PSM was quickly moved to affect cancer cells’ growth. The experiments in triplicate was dependently repeated for three times. The results are shown as the mean ± sd. Student’s t-tests were performed and the significance was indicated as ***p < 0.005, **p < 0.01, *p < 0.05, respectively.

When the independent cytotoxicity of a single reactive species such as H_2_O_2_ or NO_2_^−^ or the combination of reactive species such as H_2_O_2_ with NO_2_^−^ could not explain the observed cytotoxicity of a direct CAP treatment generating the same amount of H_2_O_2_ or NO_2_^−^, the potential unknown but important CAP-originated reactive species were proposed to explain these puzzles^20^. We proposed these puzzles might be due to the sensitization of the CAP-activated cancer cells to even so-named safe chemicals with a safe dose. We found that the CAP-activated cells were not only sensitive to H_2_O_2_, but also to other reactive species. Here, we further demonstrated the cytotoxicity of NO_2_^−^ and NO_3_^−^ on the CAP-activated PA-TU-8988T cells. It is found that NO_2_^−^ (50 μM) and NO_3_^−^ (50 μM) did not have any cytotoxicity on the PA-TU-8988T cells without an activation due to the CAP treatment (Fig. 5a). However, after the activation of CAP treatment, the NO_2_^−^ with a safe dose (50 μM) were also obviously toxic to PA-TU-8988T cells (Fig. 5a). The cytotoxicity of NO_3_^−^ did not significantly affected by the activation of PA-TU-8988T cells by CAP treatment (Fig. 5a).

Other chemicals or reactive species are also formed in the CAP-stimulated medium (PSM). Thus, we further investigated the cytotoxicity of PSM on the CAP-activated cells. We intentionally treated DMEM with a relatively short time length to decrease the cytotoxicity of PSM on the cancer cells without activation. The concentration of H_2_O_2_ in PSM was shown in Supporting Materials, Fig. S2b. PSM was used to affect the growth of cancer cells with or without the CAP activation, following the protocols illustrated in Fig. 3. It is found that PSM could cause much stronger cytotoxicity on the CAP-activated PA-TU-8988T cells than that on the PA-TU-8988T cells without a CAP treatment (Fig. 5b). This result clearly explained the puzzle that a direct CAP treatment causes a much stronger cytotoxicity over the cancer cells compared with an indirect CAP treatment did^16,17^. The data of the Case 1 and Case 2 in this experiment were shown in Supporting Materials, Fig. S3.

**Figure 6:**
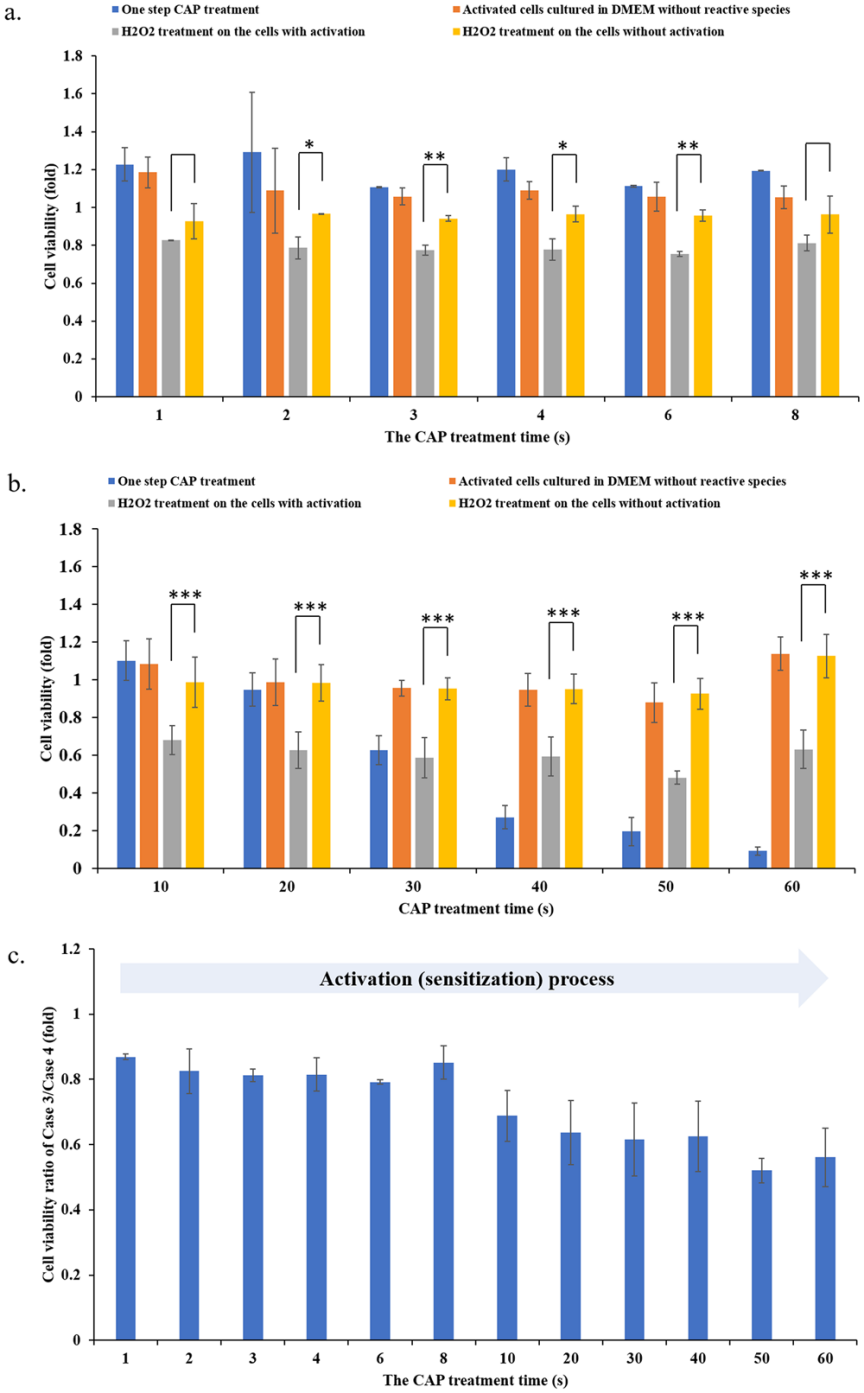
The activation starts at the very early stage of CAP treatment. (**a**) 1 s to 8 s of CAP activation. (**b**) 10–60 s of CAP activation. (**c**) The calculated cell viability ratio of Case 3/Case 4. Since the initial 10–20 s during the CAP treatment, the cells would have observable activation state. The longer CAP treatment, the stronger activation effect. The experiments in triplicate was dependently repeated for at least three times. The results were shown as the mean ± sd. Student’s t-tests were performed and the significance was indicated as ***p < 0.005, **p < 0.01, *p < 0.05, respectively.

Furthermore, we investigated the whole-time length of the activation state. We first investigated the minimum CAP treatment time that would be enough to sensitize the cancer cells. According to the protocols illustrated in Fig. 3, the CAP treatment was first performed for 1 s, 2 s, 3 s, 4 s, 6 s, and 8 s. The CAP treatments with these time lengths have been investigated through comparing the sensitization of cancer cells to H_2_O_2_ (22 μM) treatment. Comparing the data of the activated cells (gray bar) versus the non-activated cells (yellow bar) in Fig. 6a, we identified that as short as 2 s of CAP treatment could also sensitize the cancer cells to H_2_O_2_ at a relative weak level. The 10 s, 20 s, 30 s, 40 s, 50 s, and 60 s of CAP activation was also performed on cancer cells. Using the same protocols illustrated above, we found that the activation effect on these cancer cells became clear when the CAP treatment time was longer than 10 s (Fig. 6b). The observation of the activation state was based on the comparison between the data of Case 3 (gray bars) and Case 4 (yellow bars). We further calculated the cell viability ratio of Case 3/Case 4 and was shown in Fig. 6c, which would more clearly demonstrate the evolution of the activation on cells. The cell viability ratio of Case 3/Case 4 would decrease from 1 to a smaller value, once the activation state could be observed. The general trend was that the longer CAP treatment formed a stronger activation effect. Based on these observations in Figs 4 and 6, we proposed that the activation on PA-TU-8988T cells would have at least two stages: a slow activation stage in the initial 10 seconds during the CAP treatment and a fast activation stage after the initial 10 seconds of the CAP treatment.

**Figure 7:**
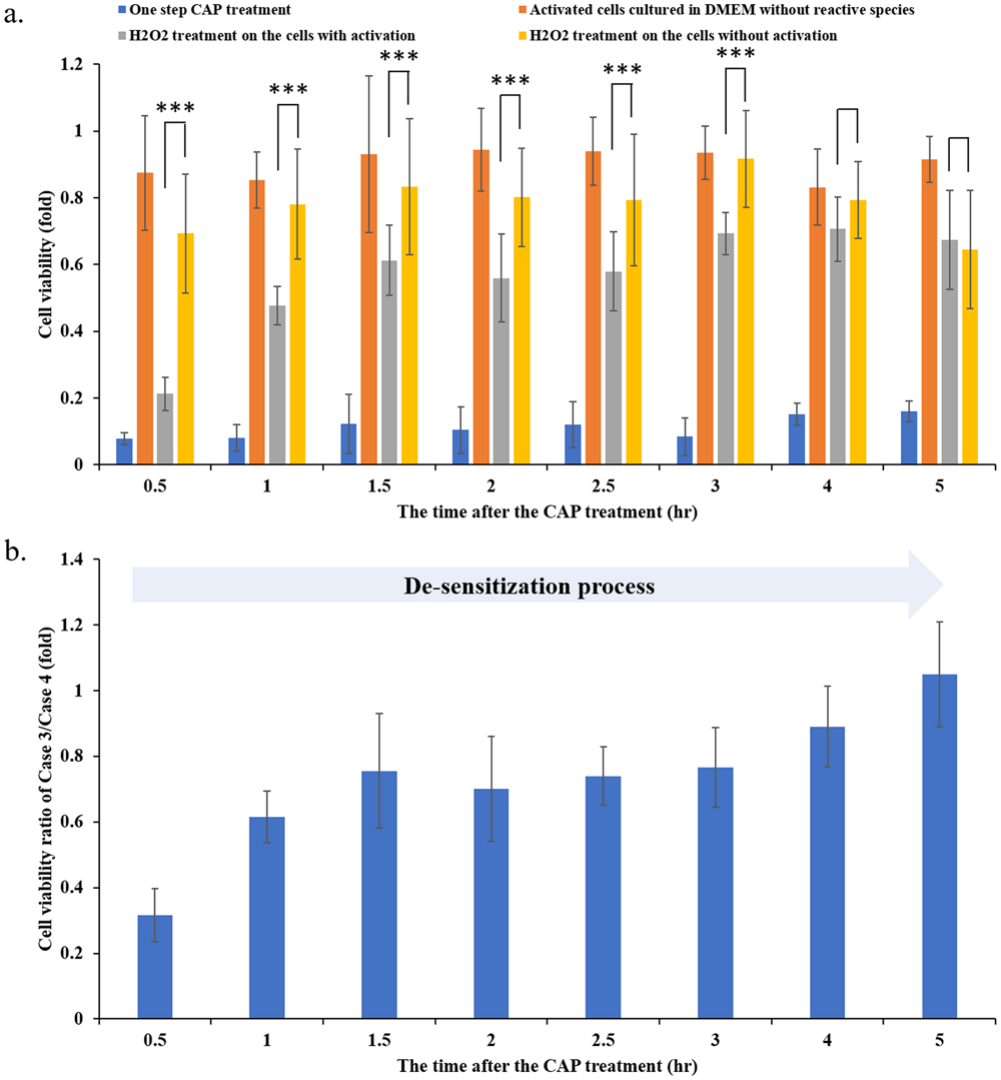
The de-sensitization of the CAP-activated cancer cells lasted 5 hours after a 1 min of CAP treatment. (**a**) The evolution of the activation state after the CAP treatment. (**b**) The calculated cell viability ratio of Case 3/Case 4. The 22 μM of H_2_O_2_ treatment was used as a probe to detect the existence of the activation state after a 1 min of CAP treatment. All protocols were the same as we introduced in Fig. 3. The data of Case 1, Case 2, Case 3, and Case 4 were shown in blue, orange, gray, and yellow, respectively. The H_2_O_2_ treatment was performed at a specific time after the CAP treatment, such as 0.5 hr, 1.0 hr, 1.5 hr, etc. The experiments performed in triplicate have been independently repeated for at least 3 times. The results were shown as the mean ± sd. Student’s t-tests were performed and the significance was indicated as ***p < 0.005, **p < 0.01, *p < 0.05, respectively.

In contrast, the de-sensitization of the CAP-activated cancer cells lasted much longer than the sensitization process. We further investigated the evolution of the activation state of cancer cells after the CAP treatment. The protocols were illustrated in Supporting Materials Fig. S4. The H_2_O_2_ treatment was also used as the probe to detect the existence of the activation state. The CAP jet was still used to treat PA-TU-8988T cells first. Then, the medium was quickly (<20 s after the CAP treatment) removed and renewed by the new DMEM. Different from the previous protocols shown in Fig. 3, H_2_O_2_ were not immediately added into DMEM to affect cells. We added H_2_O_2_ (22 μM) into DMEM every 30 minutes after the CAP treatment. Ultimately, these cancer cells were cultured for 3 days prior to the cell viability assay. The results were shown in Fig. 7a. To facilitate the understanding on these data, the ratio between the cell viability of Case 3 and the cell viability of Case 4 was calculated and was shown in Fig. 7b. A full de-sensitization meant that a H_2_O_2_ treatment would not cause stronger cytotoxicity on the cancer cells with activation than the cancer cells without activation. The complete de-sensitization meant that the cell viability ratio of Case 3/Case 4 was 1. Based on this definition, it is found that the whole de-sensitization process lasts 5 hours after the CAP treatment. The de-sensitization process had two stages. Over the first hour after the CAP treatment, there was a quick de-sensitization process. Another stage was a slow de-sensitization stage, starting from the initial first hour to the fifth hour after the CAP treatment.

## Discussions

In plasma medicine, a basic concept is the role of CAP during the CAP treatment. This is the foundation of understanding any cellular responses to a CAP treatment. The previous studies mainly focused on the long-term effect of CAP treatment, which was mainly about the cellular response several hours or even several days after a CAP treatment^21^. These responses include cellular damage due to the rise of intracellular ROS, such as the damage on the cellular membrane and membrane proteins, the damage on the extracellular matrix, damage to the genome, the mitochondrial damage, as well as the cell cycle arrest^7^. The death of cancer cells is mainly due to the CAP-triggered apoptosis, though necrosis and autophagy have also been observed^22,23^.

These cellular responses to CAP treatment are thought to be just due to the effect of long-lived reactive species. Generally, ROS may play a more important role than RNS in the cytotoxicity of the CAP treatment on cancer cells. The CAP-originated H_2_O_2_ has been regarded as the major reactive species causing the death of many cancer cell lines *in vitro*^24^. However, in view of results presented in this paper and other previous observations, this conclusion is not completely accurate. For example, a CAP treatment can still result in noticeable cytotoxicity on H_2_O_2_-resistant cell lines such as melanoma cells. Clearly, other factors during the CAP treatment also play the dominant role in some cases. Scientists in plasma medicine tend to assume some unknown reactive species may just be the other factors mentioned here.

The short-term or the instantaneous cellular responses to the CAP treatment has been completely neglected for years in plasma medicine. We defined the instantaneous response to being the response occurring within several seconds to minutes after the CAP treatment. The discovery of the cell-based H_2_O_2_ generation was the first experimental evidence about such instantaneous cellular response to a CAP treatment^16^. Many cancer cell lines can generate micromolar (μM) level H_2_O_2_ during just a 1 min of CAP treatment^16^. The activation state of cells is another instantaneous cellular response to a CAP treatment. As revealed in this study, the observable activation state can be observed even just 2 s during the CAP treatment (Fig. 6b). The activation state is obviously present about 10–20 s into the CAP treatment (Fig. 6a). In contrast, the de-sensitization process of the activated cancer cells is much longer than the sensitizing process. The cancer cells will gradually lose their sensitivity to H_2_O_2_ due to the slow loss of the activation state over the initial 5 hours post the CAP treatment (Fig. 7). The quick sensitization and the slow de-sensitization are the unique features of the CAP activation. But, we need to point out that most ROS including H_2_O_2_ will be consumed by PA-TU-8988T cells in the initial 3 hours after the CAP treatment^10,15^. Thus, if the whole de-sensitization process lasts more than 3 hours, its contribution to the cytotoxicity of CAP treatment *in vitro* may be not so strong, because of the clearance of ROS in the medium. However, such a long and slow de-sensitization process may have important biological impact *in vivo*. In addition, we need to emphasize that we just investigated the de-sensitization process occurring in the new DMEM without reactive species. In the direct CAP treatment, the CAP-originated reactive species exist in the extracellular environment over at least several hours after the treatment. Whether these reactive species will affect the de-sensitization is still unknown.

Our discovery did not overturn the commonly accepted conclusion about the key role of reactive species in this field. Our finding just demonstrated that the previous understanding was somewhat a simplified picture. In our new view of the anti-cancer mechanism of CAP treatment, CAP plays two important roles *in vitro* (Fig. 1). The first role is providing abundant reactive species in the extracellular environment. These reactive species need an accumulation time such as several minutes to reach a relatively high concentration to exert an observable effect on cancer cells. For RNS such as NO_2_^−^ and NO_3_^−^, the cytotoxicity on some cell lines will not be observed even though their concentrations are as high as 1 mM^16^. Due to the consumption by cells, at least ROS such as H_2_O_2_ will only exist in the medium for several hours after a CAP treatment^16^. The CAP treatment will be regarded as a simple chemical treatment based on reactive species if we just consider the first role mentioned here. Clearly, the CAP-treated medium mainly affects cells via this mechanism.

The unique feature of CAP treatment relies on its second role, that is activating the cancer cells during the direct CAP treatment. As we revealed in this study, the activation of cells drastically decreases the threshold of these cancer cells to the cytotoxicity of several ROS and RNS. The chemical effect of these reactive species has been drastically magnified through the sensitizing cancer cells to these reactive species. For example, 50 μM NO_2_^−^ can cause strong inhibition on the growth of the CAP-activated cancer cells. On the contrary, 50 μM NO_2_^−^ cannot cause observable growth inhibition on the same cancer cell line without an activation. The activation state of cells also direct demonstrates that even some safe chemicals such as RNS will also be toxic to the cancer cells during the CAP treatment. Similar analysis has been neglected in all previous references.

Based on these results, a direct CAP treatment definitively displays stronger cytotoxicity over cancer cells compared with an indirect CAP treatment (Figs 1 and 2a). Furthermore, the activation effect of CAP treatment is a fundamental difference between CAP treatment and other common chemical treatments. We still do not know the essence and the underlying mechanism of such an activation state based on CAP treatment. It may be due to the activation of specific pathways or the expression of specific proteins in the CAP-treated cells. The activation may be also due to the instantaneous physical change on the CAP-treated cells. Thus, there are many questions that need to be answered in the future through systematically analyzing the instantaneous change on cells due to CAP treatment.

## Conclusions

In this study, through the demonstration of the activation state of the pancreatic carcinoma cell line PA-TU-8998T after the direct CAP treatment, we provided a new perspective to understand the basic question about the CAP cancer treatment. A CAP treatment plays at least two important roles in its cytotoxicity on cancer cells. One is activating the cancer cells into a sensitive state, in which the cancer cells become sensitive to ROS and RNS, including H_2_O_2_ and NO_2_^−^. However, the activation on these cells will not cause the noticeable growth inhibition or cell death without the presence of reactive species in the extracellular environment. The activated cells will gradually de-sensitize over the initial 5 hours after the CAP treatment. The quick sensitization and the slow de-sensitization are the two basic features of the activation state on the CAP-treated cancer cells. The accumulation of the reactive species in the extracellular environment is another important role of CAP treatment on cancer cells. Due to the activation on cancer cells, the reactive species with a low concentration will also able to cause strong cytotoxicity. In addition, these reactive species alone will not cause observable damage on the cancer cells without such activation. Further research looks to analyze this activation state of cancer cells as we look to better understand the anti-cancer mechanism of CAP treatment.

## Electronic supplementary material

Figure S1, Figure S2, Figure S3, and Figure S4.
