# Supplementary material for: The Cell Activation Phenomena in the Cold Atmospheric Plasma Cancer Treatment
Source: Sci Rep. 2018 Oct 18;8:15418. doi: 10.1038/s41598-018-33914-w (PMC6194007; doi:10.1038/s41598-018-33914-w)
Supplement: Supplementary file 1 — Figure S1, Figure S2, Figure S3, and Figure S4. [file 41598_2018_33914_MOESM1_ESM.docx]

**The Cell Activation Phenomena in the Cold Atmospheric Plasma Cancer Treatment**

Dayun Yan^1,^*^,§^, Wenjun Xu^2,^*, Xiaoliang Yao^1^, Li Lin^1^, Jonathan H. Sherman^3^, Michael Keidar^1,§^.

^1^. Department of Mechanical and Aerospace Engineering, The George Washington University, Science & Engineering Hall, 800 22^nd^ Street, NW, Washington, DC, 20052, USA.

^2^. State Key Laboratory of Electrical Insulation and Power Equipment, Xi’an Jiaotong University, Xi’an, 710049, China.

^3^. Neurological Surgery, The George Washington University, Foggy Bottom South Pavilion, 22^nd^ Street, NW, 7^th^ Floor, Washington, DC, 20037, USA.

* These authors contributed equally to this manuscript.

§ Corresponding authors: Dayun Yan, [ydy2012@gwmail.gwu.edu](mailto:ydy2012@gwmail.gwu.edu).

Michael Keidar, [keidar@gwu.edu](mailto:keidar@gwu.edu).


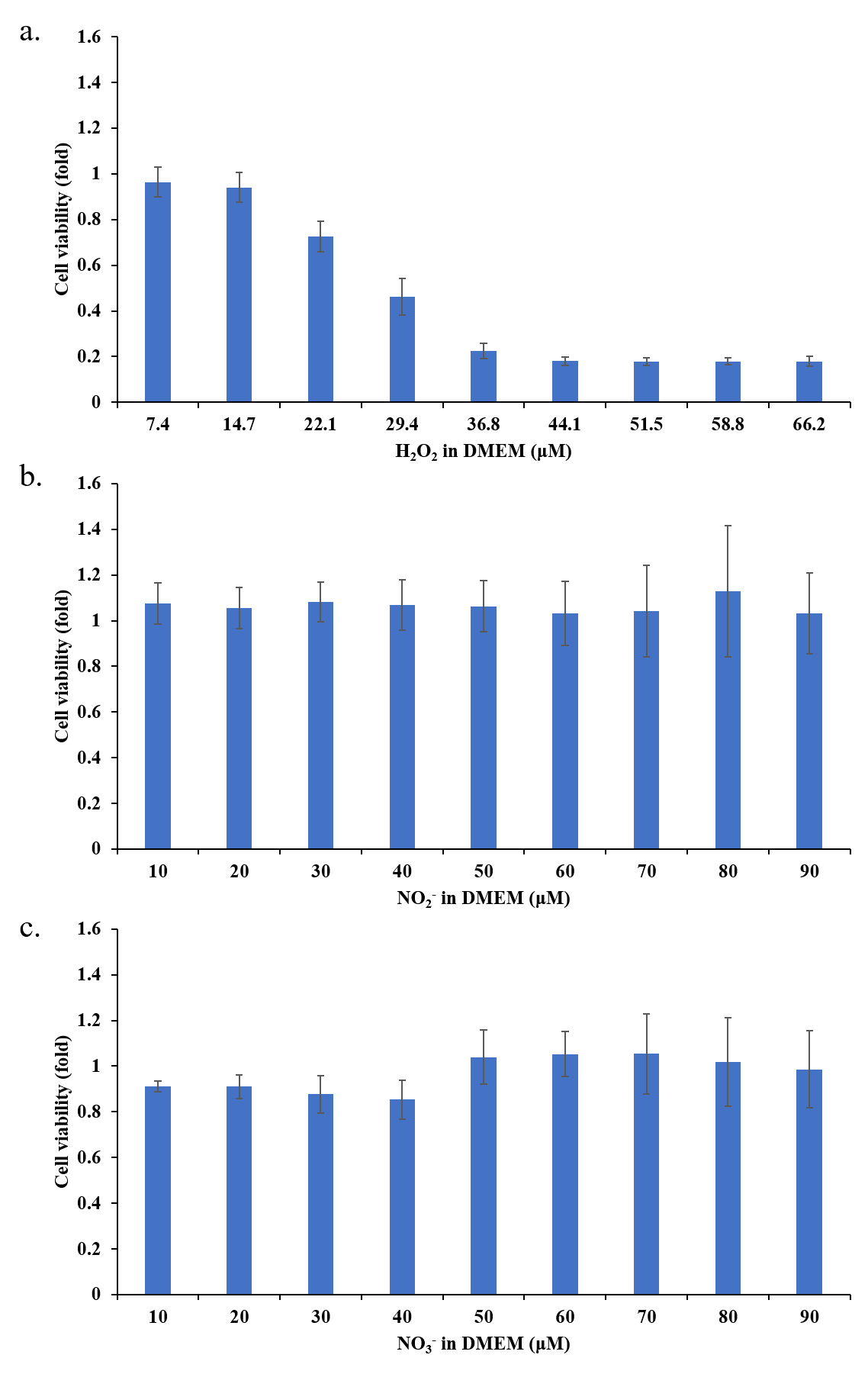


**Supporting Materials. Fig. S1.** **The cytotoxicity of H_2_O_2_,** **NO_2_^-^, and NO_3_^-^ on PA-TU-8988T cells.** (a) H_2_O_2_ treatment. (b) NO_2_^-^ treatment. (c) NO_3_^-^ treatment. The H_2_O_2_ with a relative low concentration will cause a noticeable killing effect on cancer cells. In contrast, the NO_2_^-^ and NO_3_^-^ with a relative high concentration (90 μM) are still safe for PA-TU-8988T cells. All experiments were performed in sextuplicate and were independently repeated for at least three times. The results are shown as the mean ± sd.


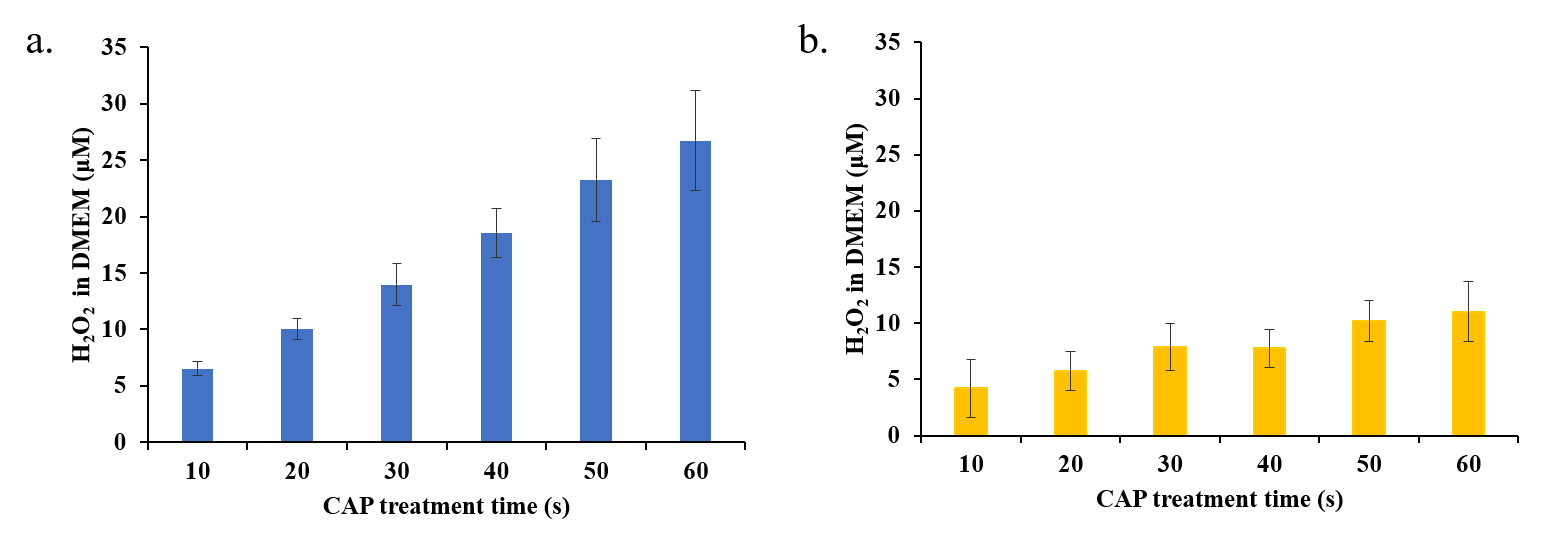


**Supporting Materials Fig. S2.** **The H_2_O_2_ generation in the CAP-treated DMEM.** (a) 55 μL/well DMEM in a 96-well plate. (b) 1000 mL/well DMEM in a 12-well plate. The H_2_O_2_ concentration in the CAP-treated medium were measured by using Fluorimetric Hydrogen Peroxide Assay Kit (Sigma-Aldrich, MAK165). The operations were performed according to the protocols provided by manufacturers. The fluorescence was measured using an H1 microplate reader (Hybrid Technology) at 540/590 nm. The final fluorescence of the experimental group was obtained by deducting the fluorescence of the control group from the fluorescence of the experimental group. H_2_O_2_ concentration were calculated based on the standard curve. The experiments in triplicate was dependently repeated for twice. The results are shown as the mean ± sd.


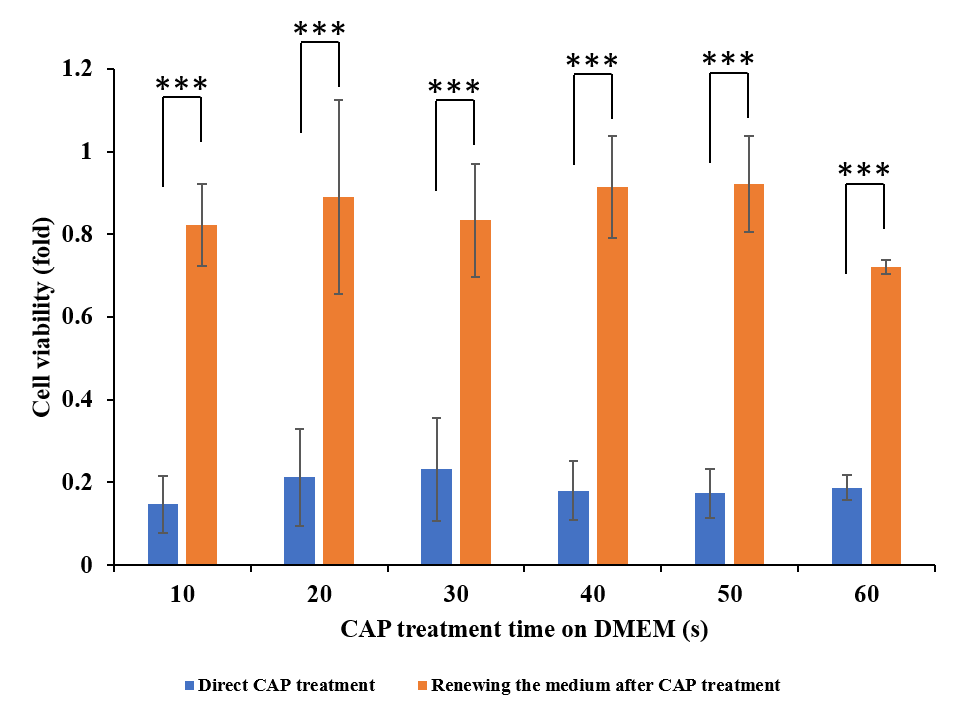


**Supporting Materials. Fig. S3. The data for the Case 1 and Case 2 in Fig. 5b.** The experiments in triplicate was dependently repeated for at least three times. The results are shown as the mean ± sd. Student’s t-tests were performed and the significance was indicated as *** p<0.005, ** p<0.01, * p<0.05, respectively.


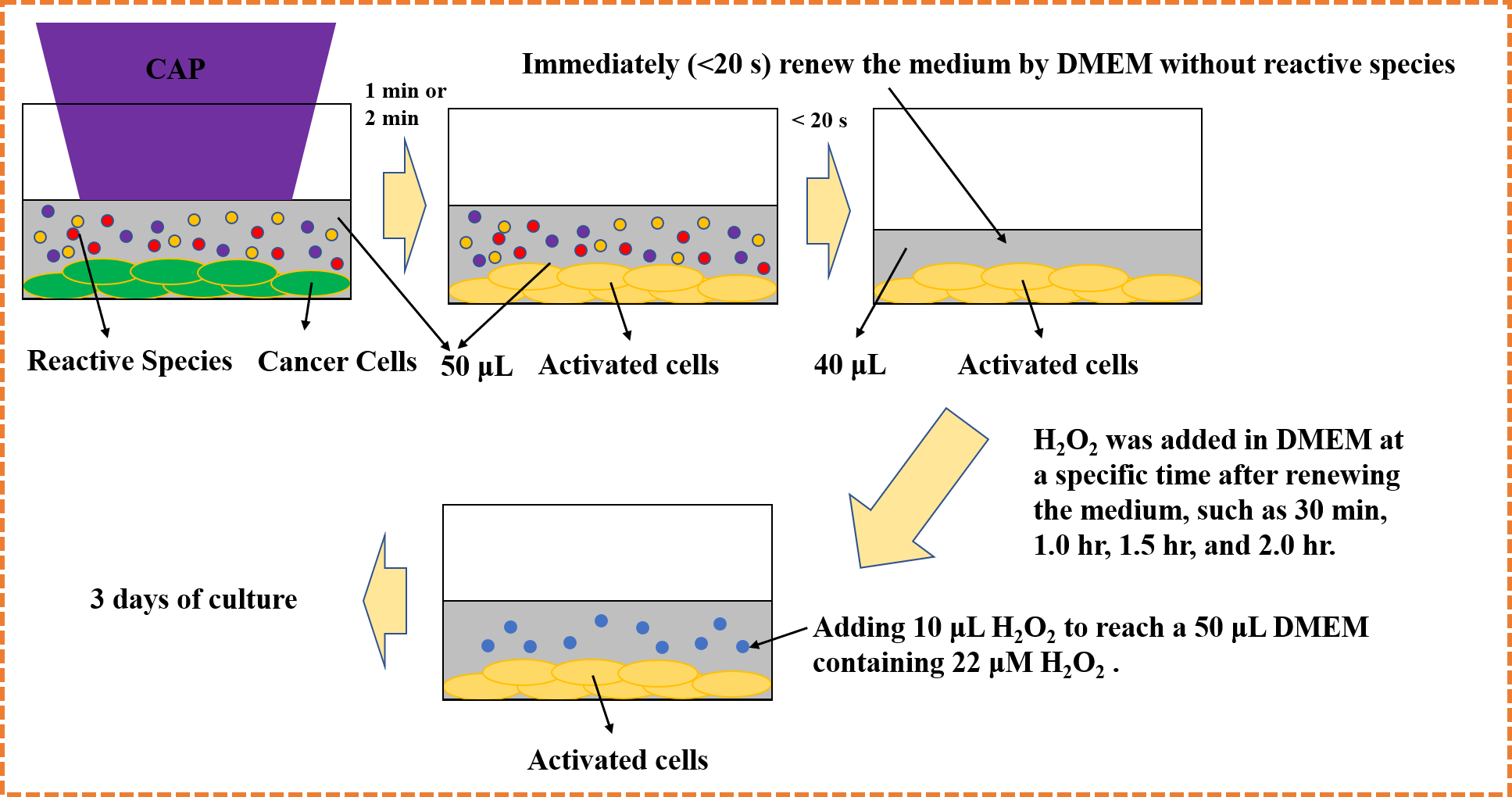


**Supporting Materials Fig. S4. The protocols to investigate the de-sensitization of the activated cancer cells.** Different from the experiments in Fig. 3, Fig. 4, Fig. 5, and Fig. 6, H_2_O_2_ did not immediately affect the growth of the CAP-activated (treated) cancer cells. The CAP-activated cancer cells were first cultured in the renewed DMEM for a specific time, such as 0.5 hr, 1 hr, 1.5 hr, 2 hr, etc. After that, H_2_O_2_ was added in DMEM to affect the growth of cancer cells.
